# Research Progress on Neural Cell Culture Systems

**DOI:** 10.2174/011570159X360193250219082312

**Published:** 2025-03-12

**Authors:** Ting Li, Xiaosong Qin, Qiang Ao

**Affiliations:** 1 Department of Laboratory Medicine, Shengjing Hospital of China Medical University, Shenyang, China;; 2 Liaoning Clinical Research Center for Laboratory Medicine, Shenyang, China;; 3 Department of Tissue Engineering, School of Intelligent Medicine, China Medical University, Shenyang, China;; 4 NMPA Key Laboratory for Quality Research and Control of Tissue Regenerative Biomaterial & Institute of Regulatory Science for Medical Device & National Engineering Research Center for Biomaterials, Sichuan University, Chengdu, China

**Keywords:** Multi-dimensional culture systems, neural cell, *in vitro*, neuronal disease, repair, regeneration

## Abstract

The nervous system, including the central nervous system and peripheral nervous system, has the most intricate structure and function among all systems in the human body. In studies of physiological and pathological functions, cell culture systems serve as an indispensable tool to simulate the nervous system *in vivo*. Two-dimensional (2D), three-dimensional (3D), and four-dimensional (4D) neural cell culture systems are used to assess the functional interconnectivity of neuronal tissues and have markedly advanced in recent years. Although 2D culture systems have predominated, they cannot accurately recapitulate the dynamic complexity of the *in vivo* environment, cell-cell communication, and nervous system structures. Consequently, studies have shifted to using 3D or 4D cell culture systems to achieve more realistic biochemical and biomechanical microenvironments. Nevertheless, many limitations persist in 3D or 4D culture systems, including difficulties in deciphering dynamic and reciprocal remodeling processes, as well as the spatiotemporal distributions of oxygen, nutrients, and metabolic waste. Here, we review 2D, 3D, and 4D culture systems, discuss the advantages and limitations of these techniques in modeling physiologically and pathologically relevant processes, and suggest directions for future research.

## 1 INTRODUCTION

The nervous system includes the central nervous system (CNS) and the peripheral nervous system. In terms of structure and function, it is the most intricate system in the human body. The fundamental component of the nervous system is nervous tissue, which consists primarily of neurons, Schwann cells (SCs), and glial cells, as well as other elements. Animal models are considered the most suitable method to simulate the nervous system *in vivo* to study its physiological and pathological functions [1]. However, in animal models, only the histiocytomorphology of nervous systems can be observed after sacrifice, and the interactions between various cell types constituting the tissue cannot be observed in real-time [2, 3]. Therefore, to advance the study of the mechanisms underlying neural cell behavior and the neurotoxic effects of chemicals *in vivo*, neuroscientists and biologists have developed *in vitro* systems to culture nerve cells [4, 5]. These systems, including two-dimensional (2D), 3D, and 4D neural cell culture systems have markedly improved to assess the functional interconnectivity of neuronal tissues in recent years.

The 2D cell culture system refers to the growth of cells on a flat substrate, such as a Petri dish, microscope slide, or culture plate. The cells can extend only along a plane during the culture process with routine cell culture techniques. Systems of 2D culture are widely applied in basic research, drug discovery, biotechnology, and the production of specific biological products, including monoclonal antibodies and recombinant proteins [6-8]. Moreover, the use of induced pluripotent stem cells (iPSCs) in 2D culture systems can aid in understanding neurotransmission and the differentiation of neurons, astrocytes, and microglia, thus providing insights into the genetic and molecular conditions underlying neurological disorders [9, 10]. Unfortunately, although conventional 2D culture systems can use human cells to avoid inter-species differences, these models are too simple, particularly in terms of structure, to recapitulate the complex nervous system. The development of 3D culture systems has enabled the use of self-organizing stem cells to fabricate organoids (mini-organs in culture dishes) that recapitulate the cellular heterogeneity, structure, and functions of the corresponding organs [11, 12].

**Fig. (1):**
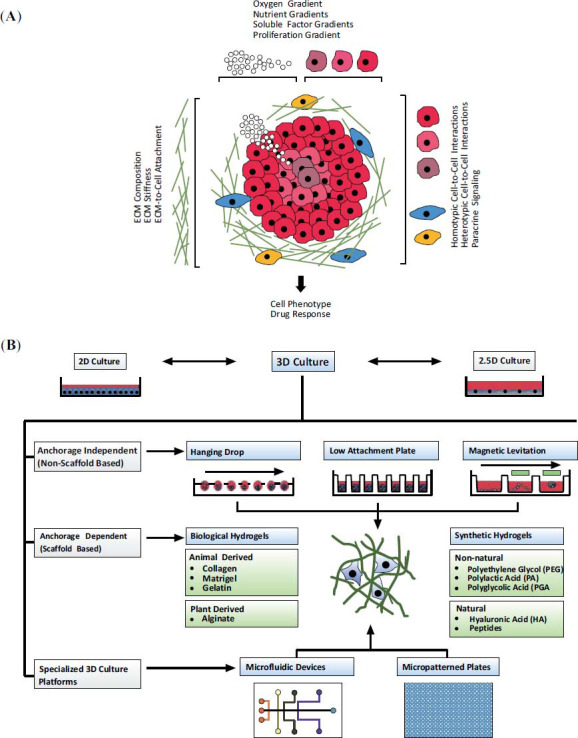
Various types of 3D culture systems (**B**) have been designed based on the characteristics of cells and their microenvironment (**A**). See a more detailed description in Section 3. This figure is adapted with a few modifications from the open access paper: Langhans S.A. Three-Dimensional *in Vitro* Cell Culture Models in Drug Discovery and Drug Repositioning. Frontiers in pharmacology, 2018, 9, 6.

In recent years, 3D cell culture systems have evolved to allow the study of cell-cell, cell-matrix, and cell-microenvironment interactions that closely mimic the *in vivo* microenvironment (Fig. **1**) [13-15]. Current 3D culture systems include hydrogel-based systems using poly (ethylene glycol) (PEG) or Matrigel [16-18], spheroids [19], organoids [20], microfluidic systems [21], and organs-on-chips [22]. They exhibit similarities to *in vivo* tissue architecture, including phenotypic and functional characteristics, thereby circumventing a limitation of monolayer cultures [23, 24]. These features provide insights regarding organ behavior and bridge the gap between 2D culture systems and animal models [25]. Differentiation time course experiments have revealed that cells in 3D culture survive longer than cells in 2D culture [26]. The 3D culture technique highlights the “time” factor during the 3D culture process—a principle of “4D” biology [27, 28]. In contrast to simply seeding cells in a 3D system, 4D cell culture requires prolonged culture times and sufficient interactions between the seeded cells and the surrounding microenvironment, including the scaffold and neighboring cells, to form a specific niche favorable for resident cells [28-30]. Therefore, 4D cell culture technology has opened many new avenues for investigations of multiple components involved in the extracellular regulation of biological processes, such as neural cell proliferation, differentiation, and migration.

This review highlights recent advances related to neural cell interactions in neural cell culture based on 2D, 3D, and 4D culture systems. It discusses the advantages and limitations of these cultural systems as well as their applications and future directions in disease models.

## 2 CURRENT 2D NEURAL CELL CULTURE SYSTEMS

The 2D culture system is a traditional cell culture method that refers to the basis of various culture systems [6, 25, 31-33]. Due to its simplicity and ease of operation, 2D culture systems are widely used in various neuronal studies, such as cytological studies, drug screening, cell biology, and other fields. For example, to evaluate the biological function of palladium-reduced graphene oxide nanocomposites, the primary *in vitro* 2D culture of mouse hippocampus neurons has analyzed the neuronal morphology, including both the average length and complexity index of the neuronal neurites [34]. In addition to primary cells, 2D culture systems are highly suitable for culturing a wide range of cell types, including stem cells, cancer cells, and cell lines. They also facilitate the differentiation of specific cell sub-types *in vitro* [35-37]. The use of cell lines (*i.e*., SH-SY5Y), primary neuronal and neural culture, dissociated culture, or iPSCs culture is still widespread in neuroscience. It offers notable advantages in the investigation of some aspects of the nervous system.

However, the 2D culture system is insufficient to comprehensively evaluate the recovery of neurological function. Although researchers have attempted to reproduce the *in vivo* microenvironment under 2D conditions, cells in 3D culture systems have spatial structures that can better reveal cell behavior *in vivo* [38]. Therefore, multiple 3D cell culture systems have been developed in the field of nervous system research recently.

## 3 CURRENT 3D NEURAL CELL CULTURE SYSTEMS

3D cell culture is a type of cell culture technology that uses bioreactors to grow cells in suspension. According to the use of scaffolds, the 3D culture system was divided into a scaffold-free system and a scaffold-based system, which can form cell spheroids or organoids, respectively. With the development of technology, the 3D cell culture systems combined with microfluidic or chip technology have developed microfluidic systems and organs-on-chips.

### 3.1 Scaffold-free 3D Culture Systems

**Fig. (2):**
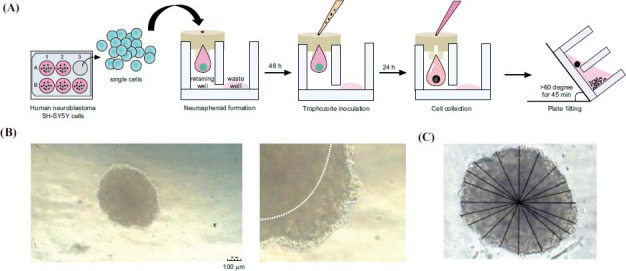
(**A**) Neurospheroid fabrication methods in a scaffold-free 3D culture system. The morphology (**B**) and diameter of human neurospheroids formed in this 3D culture system are measured (**C**). This figure is from the open-access paper: Whangviboonkij, N.; Pengsart, W.; Chen, Z.; Han, S.; Park, S.; Kulkeaw, K. Phenotypic assay for cytotoxicity assessment of Balamuthia mandrillaris against human neurospheroids. Frontiers in microbiology, 2023, 14, 1190530.

In 3D culture systems, a single neural cell type or multiple cell types may form aggregates called neurospheroids [39-44] (Fig. **2**). These neurospheroids can be generated by culturing cells in non-adherent conditions, such as suspension cultures, hanging drops, or microfluidic devices. Spheroids have been shown to deposit an intercellular ECM, whereas, in 2D culture systems, ECM may diffuse into the medium instead of maturing and accumulating [45]. Spheroids can be used to observe phenomena of growth/proliferation, drug screening, invasion, matrix remodeling, angiogenesis, and immune interactions [46-50]. For example, neurospheroids have been applied to investigate microglia migratory behaviors and activation in the engineered 3D inflammatory microenvironment in a high throughput manner not easily achievable in 2D neuronal cultures or animal models [51].

### 3.2 Scaffold-based 3D Culture Systems

**Fig. (3):**
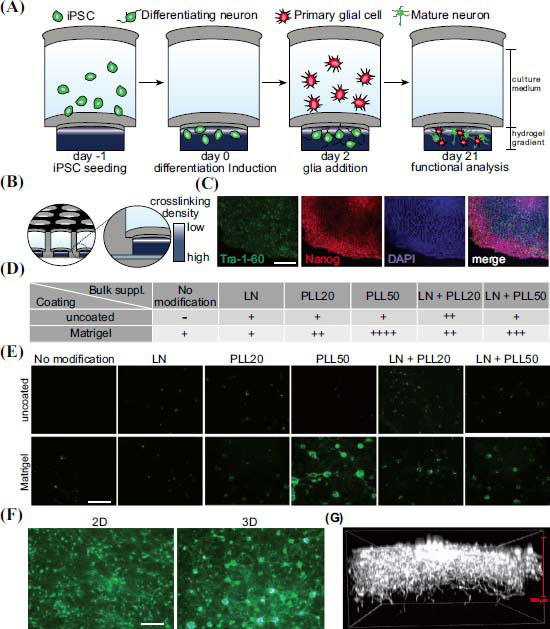
Induced pluripotent stem cell (iPSC)-derived neurons could be improved to differentiate in a 3D culture system using hydrogels as cell scaffold materials. (**A**) Illustration and timeline of the experimental paradigm. (**B**) Schematic representation demonstrating the structure of the 96-well pre-casted hydrogel cell culture plate. (**C**) Representative images of the iPS cell line used in this study. IPSCs expressed the pluripotency markers Nanog and Tra-1-60. (**D**) Table illustrating the analyzed cell culture conditions of 3D hydrogels at DIV21. Wells were either coated with Matrigel or uncoated. Hydrogel composition was modified by incorporating laminin and/or poly-L-lysine. (**E**) Representative confocal images of EGFP-expressing neurons at DIV21, cultured under the conditions mentioned in (**D**). (**F**) Representative live fluorescence images of EGFP-expressing neurons at DIV21. (**G**) Three-dimensional reconstruction of a confocal image stack showing that neurons grow to a depth of approximately 500 µm into the hydrogel at DIV21. Neurons were visualized by EGFP expression. LN: laminin; PLL50: poly-L-lysine 50 ug/mL. Scale bars: 100 µm. This figure is from the open access paper: de Leeuw, S.M.; Davaz, S.; Wanner, D.; Milleret, V.; Ehrbar, M.; Gietl, A.; Tackenberg, C. Increased maturation of iPSC-derived neurons in a hydrogel-based 3D culture. Journal of Neuroscience Methods, 2021, 360, 109254.

Hydrogels are often used as important scaffold materials in scaffold-based 3D culture systems. Hydrogels are hydrophilic polymeric materials that mimic the extracellular matrix (ECM) and have been used in 3D cell cultures and tissue engineering for several years [18, 52]. Hydrogel-based 3D culture systems can be derived from hydrogels comprising one or more natural components, such as collagen, fibrinogen, hyaluronic acid, matrigel, gelatin, and synthetic PEG hydrogels customized with cellular adhesion domains [16, 53-55]. Thermosensitive hydrogel scaffolds, as a 3D culture system, can mimic the natural ECM, thus providing a suitable environment similar to 3D tissue for cell growth, neuro-differentiation, and the transport of stem cells and drugs [56-59]. Furthermore, elevated synaptic density has been observed in iPSC-derived neurons embedded in layered, hyaluronic acid-based hydrogels [60]. One study has used BD PureMatrix hydrogels to understand the SC response in peripheral nerve injury [60]. On the basis of immunostaining, hydrogels have been demonstrated to be a valid representative ECM for culturing SCs, thus allowing neurite outgrowth to occur similarly to that observed in previous *in vivo* studies of peripheral nerve damage [61]. In addition, human neurons and glial cells have been successfully cultured in PEG-based cell cultures and found to form complex 3D structures [17]. The transcript and protein levels of dopaminergic neuronal markers are significantly higher in differentiated cells in Matrigel-based systems than in 2D culture systems [62]. Matrigel is a soluble basement membrane extracted from the Engelbreth-Holm-Swarm mouse sarcoma, a tumor rich in extracellular matrix proteins, including laminin, collagen IV, heparan sulfate proteoglycans, entactin/nidogen and so on. Choi *et al*. have shown increased maturation of human neural progenitor cell-derived neurons embedded in 0.3 to 4.0 mm-thick Matrigel [16]. Most importantly, hydrogels could allow for the internal movement of soluble factors such as cytokines and growth factors, thus supporting the long-term growth of cells [15]. These manufacturing techniques represent substantial advances in biotechnology for the development of biomimetic, replicable, and scalable *in vitro* nervous system models (Fig. **3**) [18].

The most important products performed by these scaffold-based 3D culture systems are organoids. Organoids and spheroids have several similarities and can be distinguished by their cellular organization, complexity, and functional specialization. Spheroids are simple aggregates of cells lacking distinct organization, whereas organoids are self-organized structures that resemble specific organs. Organoids are typically cultured in a specialized medium containing growth factors that promote differentiation and tissue-specific gene expression [63-66]. As a result, organoids have a complex cellular organization, spatial orientation, and function similar to that *in vivo* tissues. Organoids have been sectioned and analyzed morphologically and reported to show similar cytoarchitectures to those in the developing human cortex. Therefore, 3D organoid models can be used to study morphological changes in nigrostriatal human neurons in relation to changes in physiology [67]. Moreover, brain and spinal cord organoids have been found to recapitulate neural development *in vitro*, thus enabling exploration of the interactions among CNS regions, and the evolution of the human CNS and its unique regulatory mechanisms [68]. Importantly, organoid-based co-culture systems can be developed to study the disease processes involving multiple systems or tissues, such as neuromuscular diseases, amyotrophic lateral sclerosis, and the intricate connections between different CNS and local circuits [69]. Comparative studies have shown that findings in 3D human brain organoids support those obtained in animal or cohort studies, thus indicating that 3D human brain organoids are reliable models for evaluating developmental neurotoxicity [70]. Also, organoids are a powerful tool for cell therapy, providing new insights into the treatment of nervous system disease. Currently, human iPSC-derived midbrain organoids have been proposed as a safe and effective treatment option for Parkinson’s disease (PD), with the aim of replacing lost dopaminergic neurons and restoring motor function [71]. Although organoids have gained popularity in physiology and cell-based assay development, they also have several limitations that require improvement [72]. Organoid creation is expensive and time-consuming, and complicating factors in organoid creation may include strict reliance on growth factors and signaling gradients to ensure lineage specification and balanced stem cell renewal. These problems can be solved by using microfluidic technology [73-75].

### 3.3 Multi-technology 3D Culture Systems

**Fig. (4):**
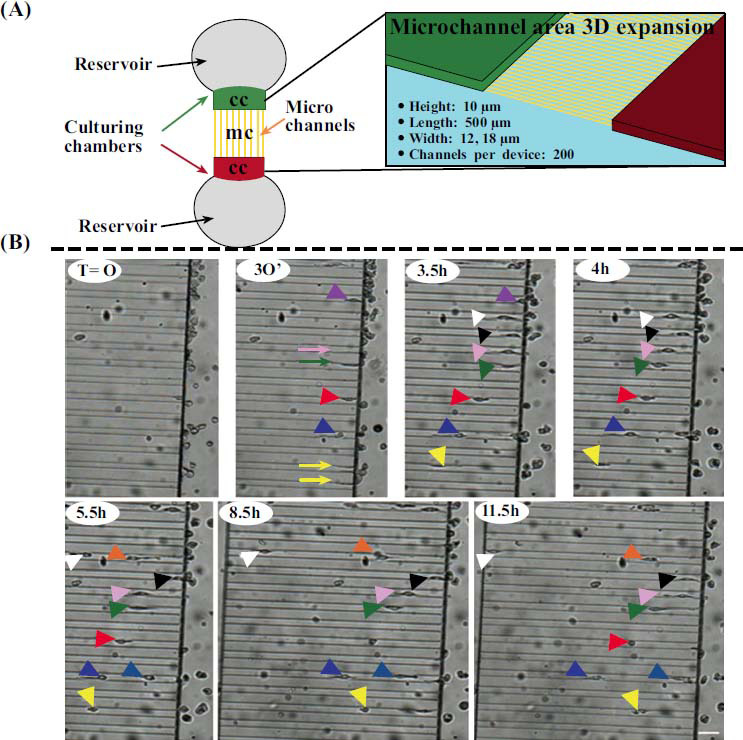
(**A**) A schematics of the microfluidic device. (**B**) Using this microfluidics, the spontaneous migration of microglia in microfluidic channels could be observed in real-time. This figure is adapted with a few modifications from the open-access paper: Cho, H.; Hashimoto, T.; Wong, E.; Hori, Y.; Wood, L.B.; Zhao, L.; Haigis, K.M.; Hyman, B.T.; Irimia, D. Microfluidic chemotaxis platform for differentiating the roles of soluble and bound amyloid-β on microglial accumulation. Scientific Reports, 2013, 3, 1823.

High-speed development of micromanufacturing technology has promoted the emergence of microfluidic systems. Microfluidics is a technology that uses channels ranging in size from tens to hundreds of micrometers (10^-9^-10^-18^ L) to precisely monitor and manipulate the cell growth microenvironment and to easily add appropriate mechanical, biochemical, or electrical stimulation [76-78]. Cells obtain nutrients and oxygen *via* the fluid circulation, and can be exposed to the spatial cues or signaling gradients necessary for differentiation, growth, viability, and proliferation [79, 80]. In recent years, microfluidic systems have been developed for a wide range of applications in cancer research, drug screening, vascular models, neuroscience, and analysis of dynamic cell-cell interactions under reproducible *in vitro* culture conditions [69, 81]. For example, microfluidics have been used to mimic the interaction of microglia with the complex brain microenvironment *in vivo* (Fig. **4**) [82-88]. Machado *et al*. have used a microfluidic system made of polydimethylsiloxane to isolate and orient the growth of axons and to co-culture oligodendrocytes and axons in the CNS [89].

**Fig. (5):**
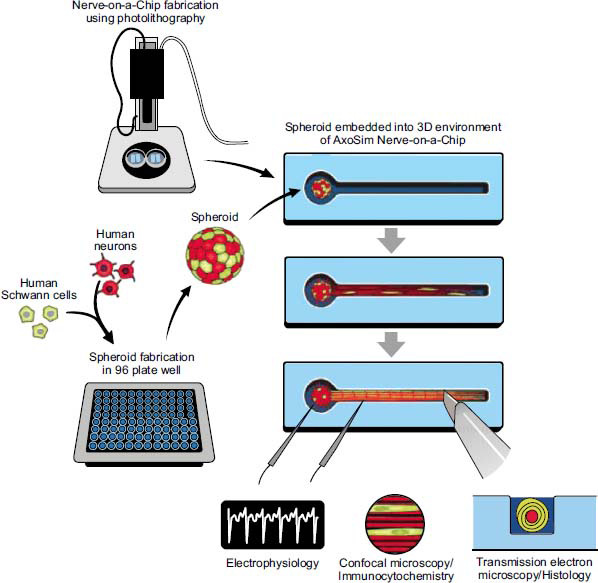
The neurospheroid is fabricated into a human nerve-on-a-chip using photolithography to simulate *in vivo* nerves for *in vitro* experiments, such as electrophysiology, immunocytochemistry, or histology assays. This figure is from the open access paper: Sharma, A.D.; McCoy, L.; Jacobs, E.; Willey, H.; Behn, J.Q.; Nguyen, H.; Bolon, B.; Curley, J.L.; Moore, M.J. Engineering a 3D functional human peripheral nerve *in vitro* using the Nerve-on-a-Chip platform. Scientific Reports, 2019, 9(1), 8921.

Organ-on-a-chip technology, which has rapidly developed in recent years, is likely to overcome most shortcomings of conventional 3D culture systems. Recent research has developed human cell-derived 3D culture systems with the nerve-on-a-chip platform (Fig. **5**) [22]. Peripheral nerve tissue-on-a-chip platforms build upon microfabrication techniques and tailor them to develop testable 3D models with physiologically relevant outcomes, notably electrophysiology. These constructs can direct axonal growth, support glial (*e.g*., myelination) and neural (*e.g*., synaptic transmission) cell functions, and record electrophysiological signals with high signal-to-noise ratios [90]. Brain-on-chip (BOC) platforms, such as nano grooves and microtunnel structures, have advanced *in vitro* neuronal models by engineering the microenvironment to enhance the maintenance, manipulation, and analysis of neural cell cultures. To study morphological aspects of neuronal differentiation, such as neuronal outgrowth (neurite) length and direction, compartmentalization, and nanotopography are strategies that have shown promise as part of the BOC toolbox [91].

## 4 CURRENT 4D NEURAL CELL CULTURE SYSTEMS

**Fig. (6):**
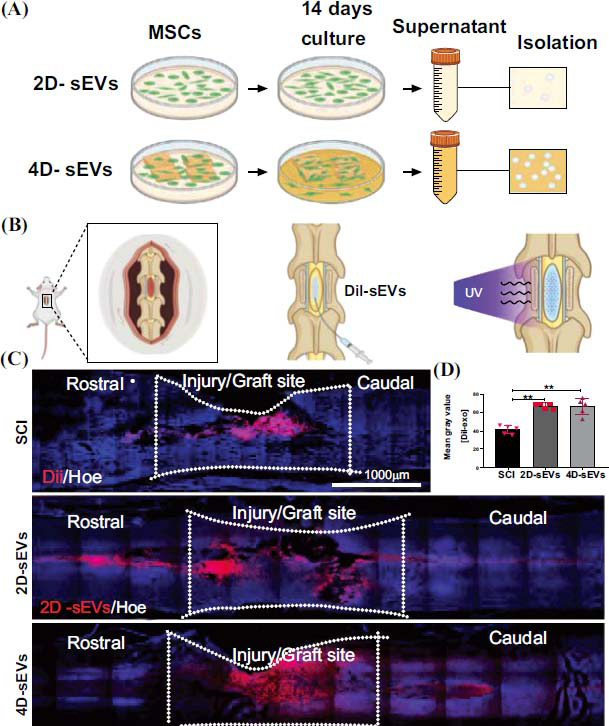
The sEVs are acquired in 2D and 4D culture systems, respectively, and used to repair damaged spinal cord. The 4D-sEVs have better repair and anti-inflammatory capacity compared to 2D-sEVs. (**A**) Flow chart demonstrating the sEVs acquisition process. (**B**) Schematic showing how sEVs are injected into the spinal cord. (**C**) Distribution and migration of sEVs in the injury/graft site of the spinal cord visualized using fluorescent images. (**D**) Bar charts showing the mean fluorescence intensity in three groups within the injury/graft site of the spinal cord. Statistic difference: **p < 0.01. This figure is from the open access paper: Wang, J.; Wei, Q.; Yang, Y.; Che, M.; Ma, Y.; Peng, L.; Yu, H.; Shi, H.; He, G.; Wu, R.; Zeng, T.; Zeng, X.; Ma, W. Small extracellular vesicles derived from four dimensional-culture of mesenchymal stem cells induce alternatively activated macrophages by upregulating IGFBP2/EGFR to attenuate inflammation in the spinal cord injury of rats. Frontiers in bioengineering and biotechnology, 2023, 11, 1146981.

Cell-laden matrices in 3D space that change over time due to physical, chemical, or biological processes, referred to as 4D biology, are being designed to mimic the functions of human tissues and organs *in vitro*. The ultimate goal of a 4D neural cell culture system is to generate microenvironments for use in therapeutic cell delivery, *in vitro* modeling of disease progression, and design of physiologically relevant models for drug discovery. Recent studies have focused on the main features of small extracellular vesicles (sEVs) derived from 4D mesenchymal stem cell culture (4D-sEVs) and their effects on regulating the inflammatory response in injured areas of the spinal cord (Fig. **6**) [28]. Compared to 2D-sEVs, 4D-sEVs have 147 up-regulated proteins and 43 down-regulated proteins and exhibit a stronger anti-neuro-inflammatory capacity, suitable for repairing trauma in the CNS, such as spinal cord injury [92]. Although the field of 4D culture systems remains in its infancy, numerous opportunities exist to pursue complex biological inquiries (such as understanding the molecular mechanisms linking ECM signals to nuclear function) and to provide practical solutions for expanding, differentiating, and delivering cells for clinical applications.

## 5 2D *VS.* 3D *VS.* 4D CULTURE SYSTEMS: ADVANTAGES AND LIMITATIONS

Each cell culture system has its own advantages and limitations (Table **1**), and understanding these differences can aid in studying the nervous system *in vitro.* The main advantage of 2D culture systems is their simplicity and ease of use, accessibility, and low cost, owing to easy setup and maintenance, thereby allowing for efficient and direct analysis of cell morphology and pathological features. However, their role in disease modeling is limited because 2D cultures can only differentiate single-cell types [9, 10, 93-96] and cannot accurately recapitulate the dynamic complexity of the *in vivo* environment, cell-cell communication, and nervous system structures [97-99]. For example, because drug screening in 2D culture systems cannot represent cells in the tissue microenvironment, the failure rates of drug discovery are high, and the levels of drug approval for market entry are low [15]. To address these limitations, numerous advancements have enhanced the capabilities of 2D culture systems. For instance, techniques such as high-content imaging and transcriptomics have greatly improved the analysis and characterization of cells in 2D culture systems [100, 101], allowing a more comprehensive understanding of cellular behaviors and characteristics.

To mimic the natural structures of tissues and the cell-cell/-extracellular environment interactions, 3D culture systems have been developed [102, 103]. Compared with 2D culture systems, emerging 3D culture systems appear to be better at inducing *in vivo* cell responses under physiological and/or pathophysiological conditions [104-106]. For example, human neural precursor cells derived from the fetal spinal cord have been incubated in 2D and 3D culture systems. The cells in the 2D culture system were found to have a spindle-shaped morphology with classic hill-and-valley growth patterns, whereas in 3D, the cells grow as clusters of undifferentiated cells and cell sheets (tissue organoids) that gradually roll up like a carpet without forming a circular cell mass [107]. Therefore, 3D culture systems, such as organoids, can be used to study drug efficacy and toxicity, providing a more realistic model for drug testing and a better understanding of how drugs interact with cells. Moreover, 3D culture systems can be used in preclinical studies to provide relevant information regarding other types of diseases and can help reduce the use of animal models [25, 108, 109]. Cells in 3D culture systems are long-lived, in some cases for months; this temporal aspect of cell culture is important for modeling diseases that develop slowly, such as PD and Huntington’s disease (HD) [110, 111]. The relatively long lifespan of cell culture systems enables convenient identification of pathological defects emerging in tissues over time.

Despite the marked improvements in 3D culture systems over conventional 2D systems, current 3D culture systems have several limitations, including poor nutrient support, a necrotic core in 3D spheres, complex and time-consuming culture conditions, low reproducibility in organoids, and the requirement for encapsulating cells in hydrogels, which prevents later monitoring of the cells [18]. With growing interest in 3D culture systems, it is expected that this field will continue to develop and provide new opportunities for nervous system research in the future [15, 25].

With the development of cell culture technology, 4D cell culture systems have opened many new avenues for investigating the multiple components involved in the extracellular regulation of biological processes. Most 3D culture systems have high water content, enable ready diffusion of small molecules, and are amenable to *in situ* imaging; however, the assessment of cell output requires innovative techniques. The high-throughput technology of 4D culture systems is capable of dynamically detecting multiple extracellular signals, which is critical for researchers to discern the combinatorial effects of signals on cell output and function. However, as experiments are translated from Petri dishes to 4D, the technology itself introduces unique analytical challenges requiring creative solutions. For example, the cellular microenvironment changes over the course of experiments, resulting in cellular deposition of matrix proteins and glycans, as well as secretion of paracrine signaling factors into the culture system, confounding experimental results. Although how the initial regulation of specific signals leads to changes in cellular mechanisms can easily be evaluated in the short term, distinguishing specific effects on cellular functions in 4D culture systems is difficult without new technologies to decipher dynamic and reciprocal remodeling processes [27]. While 4D culture systems could enhance the feasibility of spheroids for testing new treatment strategies, they are subject to the same limitations as spheroids, given their analogous construction [112]. These enormous challenges can be addressed through interdisciplinary collaborations, particularly when knowledge of the stem cell niche in biological sciences is combined with biomaterial engineering in 4D culture systems. In this way, scientists can collaboratively design relevant multifunctional and bioactive matrices, and the synthesis of injectable cell scaffolds can be integrated with clinical research in cell therapy.

## 6 FUTURE PROSPECT

Cell culture, which refers to growing cells in a controlled system outside their natural environment, is one of the most basic and important methods for *in vitro* experimental research. The main types of cellular culture include 2D, 3D, and 4D cell culture systems [113]. Although 2D culture systems allow for monolayer cell culture *in vitro*, the cells do not have the same morphology [17], proliferation [18], migration [53] gene expression [16], or differentiation [54] functions as those observed *in vivo*, thus prompting the development of 3D culture systems to bridge existing knowledge gaps [114, 115]. In contrast, 3D culture systems provide a larger space for culture expansion and have the potential to include different cell types [116]. Thus, 3D culture systems better capture the complexity of the nervous system and can provide valuable information regarding cell biology, signal transduction, cell migration, drug discovery, angiogenesis, metabolic profiling, inflammation, and apoptosis. For example, 3D bioprinting based-3D culture systems using ECM bioinks, combining SH-SY5Y and LUHMES cell lines with ECM hydrogel, or incorporating microglia together with astrocytes into the organoids have been used as excellent neurotoxic models for studying PD [117-119]. Moreover, each psychostimulant drug (methamphetamine, cocaine, ecstasy, and ketamine) may have its own specific mechanism(s) mediating its deleterious effects on the brain, as well as unique deleterious interactions with other substances that may cause or contribute to brain damage [120]. In this regard, further research using appropriate cell culture systems designed to more closely mimic the patterns of drug use observed in humans will be critical to gaining novel insights into the neurotoxic effects and brain dysfunction that psychostimulants may induce in the human brain. Furthermore, there is evidence that oxidative and nitrosative damage occurs in cerebrovascular diseases such as HD, PD, Alzheimer's disease, and stroke [121, 122]. Recent studies indicate that the vitagenes play an important role in anti-oxidative damage *via* Nrf2 signalling pathways in *Caenorhabditis elegans* (*C. elegans*) models of PD [123]. Although comparative proteomics showed that 83% of the *C. elegans* proteome could be identified with human orthologs, organoids derived from human cells or other multi-dimensional culture systems would be more realistic to explore this mechanistic question.

Currently, many problems remain to be solved in existing culture systems. For example, 3D culture systems can hinder the reproducible study of whole-brain interactions, because the diversity of cell types and the ability to produce fully mature cells in these systems do not match the complexity of the human brain [124]. Moreover, one of the greatest unmet needs in complex culture systems, such as 3D and 4D culture systems, is the lack of reliable neural markers. Multiple cell types co-express glial markers, such as S100β, which are expressed in both astrocytes and oligodendrocyte precursor cells [125]. Furthermore, distinguishing between developing and terminally differentiated populations is difficult because GFAP is expressed at different levels in radial glia, immature astrocytes, and mature astrocytes [126]. Myelin basic protein can be used as a marker for oligodendrocytes, SCs, and SC tumors, and Iba1 is expressed in microglia of the CNS and macrophages of peripheral tissues [127], thus often confounding analyses and results. Therefore, new high-level expression markers and specific antibodies are needed to clearly identify each neural cell of interest at distinct stages of development. These markers would allow scientists to answer many of the current open questions in this field, particularly regarding the developmental origins of various types of human neural cells [128].

Certain viruses, such as the Japanese encephalitis virus, can cross the blood-brain barrier and enter the CNS. Many neurotropic viruses, including poliovirus, herpes zoster virus, herpes simplex virus, and rabies virus, can penetrate the local nerve along axons. Therefore, 3D neural cell culture systems can be co-cultured with microorganisms to establish microbially infected neural disease models. For instance, Zika virus*-*infected brain organoids in a co-culture system, which can mimic neural development *in vivo* to some extent, are an ideal model to reveal the mechanism of Zika-induced microcephaly [69, 129, 130]. In addition, Zika-infected brain organoids could be used to screen drugs for the treatment of Zika viral infection. Similarly, brain organoids offer a promising tool for uncovering pathophysiological clues and potential therapeutic options for neuropsychiatric complications of Coronavirus disease 2019 [131, 132]. Moreover, hydrogel-based 3D culture systems have been found to study different strains of rabies viral infection in a more physiological environment, thus broadening understanding of rabies virus-host interactions in the CNS [133]. These studies have increased confidence that more co-cultured models can be developed to treat infectious diseases of the nervous system.

## CONCLUSION

In conclusion, 2D, 3D, and 4D neural cell culture systems are all important tools in cell biology research and biotechnology, each with distinct advantages and limitations. The selection of appropriate methods for different research purposes is important for understanding cell biology and developing new therapies. In the future, more technical advances will be required to address the limitations of existing 2D, 3D, and 4D neural cell culture systems. For examples, more multi-dimensional cell culture systems and mixed diverse experimental techniques combined cell culture systems *in vivo* or *in vitro* will update our understanding of cell culture.

## Figures and Tables

**Table 1:** Advantages and limitations of 2D, 3D, and 4D cell culture.

-	**Advantages**	**Limitations**
2D culture systems	• High reproducibility and purity • Easy quantification • Potential for long-term maintenance • Unlimited nutrient exposure • Simplified culture conditions	• Random cell distribution • Limited cell-cell interactions between specific cell types • One type of neuron • Lack of specific structure
	• Near-physiological environment and developmental process	-
	• Ability to examine the interplay among cell lineages	• Higher variability
3D culture systems	• Cell heterogeneity • Cell-cell interactions • Cellular maturation • Mixed diverse cell types • Organ-specific structure	• Lack of nutrient and oxygen access for long-term culture • Difficulty in quantifying and interpreting the data
4D culture systems	• Advantages of 3D culture systems • 3D culture systems of the space-time axis	• Difficulty in deciphering the dynamic and reciprocal remodeling processes

## LIST OF ABBREVIATIONS

2D: Two-dimensional
3D: Three-dimensional
4D: Four-dimensional
4D-sEVs: sEVs Derived from 4D Mesenchymal Stem Cells Culture
BOC: Brain-on-chip
*C. elegans*: *Caenorhabditis elegans*
CNS: Central Nervous System
ECM: Extracellular Matrix
HD: Huntington’s Disease
iPSCs: Induced Pluripotent Stem Cells
PD: Parkinson’s Disease
PEG: Poly(ethylene Glycol)
SC: Schwann Cell
sEVs: Small Extracellular Vesicles
